# A Dosimetric Study Comparing 3D-CRT vs. IMRT vs. VMAT in Left-Sided Breast Cancer Patients After Mastectomy at a Tertiary Care Centre in Eastern India

**DOI:** 10.7759/cureus.23568

**Published:** 2022-03-28

**Authors:** Saroj Kumar Das Majumdar, Adhar Amritt, Sovan Sarang Dhar, Sandip Barik, Sasanka S Beura, Tushar Mishra, Dillip K Muduly, Ashish Dash, Dillip Kumar Parida

**Affiliations:** 1 Radiation Oncology, All India Institute of Medical Sciences, Bhubaneswar, Bhubaneswar, IND; 2 Medical Physics, All India Institute of Medical Sciences, Bhubaneswar, Bhubaneswar, IND; 3 Surgery, All India Institute of Medical Sciences, Bhubaneswar, Bhubaneswar, IND; 4 Surgical Oncology, All India Institute of Medical Sciences, Bhubaneswar, Bhubaneswar, IND; 5 Pharmacology, All India Institute of Medical Sciences, Bhubaneswar, Bhubaneswar, IND

**Keywords:** intensity modulated radiotherapy, volumetric-modulated arc therapy, 3d- conformal radiation therapy, tangential intensity modulated radiotherapy, radiotherapy (rt), dosimetry plan, left breast cancer, vmat, imrt, mastectomy

## Abstract

Introduction

Post-mastectomy radiation in left-sided breast cancer in women continues to pose a significant risk to the underlying lungs and heart. This study analyzed the difference in planning target volume (PTV) coverage and dose to the organs at risk (OAR) by using three different planning methods for the same patient - three-dimensional conformal radiotherapy (3D-CRT), intensity-modulated radiotherapy (IMRT), and volumetric-modulated arc therapy (VMAT).

Material and methods

Thirty-five left-sided breast cancer patients’ post-mastectomy were included in this study, and three different plans for adjuvant radiation were created using 3D-CRT, IMRT, and VMAT. The prescribed dose was 50Gy in 25 fractions. ﻿Kruskal-Wallis analysis of variance (ANOVA) was done, followed by a pairwise t-test to establish a hierarchy of plan quality and dosimetric benefits. The plans were compared with PTV_95_, homogeneity index (HI), conformity index (CI), hotspot (V_107%_), left lung V_20Gy_, mean lung dose, heart V_25Gy_, mean heart dose, and integral dose (ID) to the body.

Results

Both VMAT and IMRT led to improved PTV_95% _coverage (95.63±1.82%, p=0.000 in VMAT; 93.70±2.16 %, p=0.000; 81.40±6.27% in 3D-CRT arm) and improved CI (0.91±0.06 in IMRT [p<0.05] and 0.96±0.02 for VMAT plans [p<0.05]) as compared to 3D-CRT (0.66±0.11), which was statistically significant on pairwise analysis. In contrast, the difference in HI and reduction in hotspots were not significantly different. Left lung V_20 _was statistically very different between the three arms with the highest values in IMRT (36.64±4.45) followed by 3D-CRT (34.80±2.24) and the most negligible value in VMAT (33.03±4.20). Mean lung dose was also statistically different between the three arms. There was a statistically significant difference in mean heart dose between the three arms on pairwise analysis. Both the inverse planning methods led to a statistically significant increase in low dose volume (V_5 _and V_10_) of the ipsilateral lung, opposite lung, and heart, and increased ID to the body excluding the PTV.

Conclusion

While both the inverse planning modalities led to increased coverage, better CI, and better HI and decreased high dose volumes in OARs, there was increased low volume irradiation of heart, lungs, and body with VMAT faring marginally better than IMRT in coverage and decreasing lung irradiation with comparable heart irradiation.

## Introduction

Breast cancer is the leading cause of cancer among females in developing countries, with most cases presenting in a locally advanced stage. It constitutes the highest number of patients seen on an outpatient basis in a developing country like India, with 178,361 new cases diagnosed in 2020, constituting 26.3% of all cancer cases [1]. Mastectomy has remained the standard of care for locally advanced breast cancer (LABC). Even with the myriad options available to oncologists worldwide, radiation plays a pivotal part in multimodality treatment to prevent local recurrences leading to improved overall survival. EBCTCG meta-analysis [2] and other trials had demonstrated that post-mastectomy radiotherapy (PMRT) provides a survival advantage to patients undergoing mastectomy even when they had at least one positive lymph node. The MA-20 trial helped determine who would benefit from Comprehensive Nodal Irradiation (CNI) along with chest wall irradiation [3]. The benefit of radiotherapy comes at the expense of the heart [4], lung [5,6], and secondary breast cancer risk [7]. The heart is one of the most vulnerable organs for radiation for left-sided breast cancer, with increasing cardiac dose linked to higher mortality [4]. Compared to three-dimensional conformal radiotherapy (3D-CRT), intensity-modulated radiotherapy (IMRT) has been widely used in the last decade, allowing optimal dose distribution according to individual anatomy. It improves dose homogeneity within the irradiated breast with heart and lung sparing. A newer technique called volumetric modulated arc therapy (VMAT) was introduced by Otto [8] in 2008. The intended dose could be delivered in a single gantry rotation and decrease the treatment time compared to IMRT.

Countries with robust screening programs encounter more early-stage breast cancers. Developing countries are more likely to see LABC cases requiring multi-disciplinary management [9]. Radiotherapy forms an integral part of the management of LABC irrespective of response to neoadjuvant chemotherapy as it improves disease-free survival even in patients having pathological complete response (pCR) [10].

To answer the question of the target volume to be treated for the post-mastectomy breast, trials like multi-centric MA.20 study [3] and Poortmans et al. [11] have concluded that CNI is the way forward. The irradiation of the internal mammary chain is a challenging aspect because of the cardiac toxicity and narrow benefit margin.

As early as 2012, Sakumi et al. [12] started treating left-sided breast cancer with VMAT, concluding with the advantage of decreased treatment time. While there are multiple studies [13,14] for breast irradiation following breast-conserving surgery (BCS), very few studies analyzed the impact of IMRT or VMAT on PMRT. Few studies [15] compared all three modalities, some compared only two modalities, but the quantum of subjects compared was relatively low to draw clinically significant conclusions [16-20].

This study is being conducted to compare the dosimetric differences between these three modalities of radiotherapy (3D-CRT, IMRT, and VMAT) to the left-sided chest wall following mastectomy and determine which modality achieves maximum dose to target volume and minimal dose to organs at risk (OAR). There was a lack of adequate studies for PMRT in the Indian context; hence this study was planned.

## Materials and methods

The Institute Ethics Committee approved the study in April 2020. Forty-five left-sided breast cancer patients attending Radiotherapy OPD were recruited in this study from May 2020 to December 2021, out of which 35 were included in the final analysis after undergoing mastectomy with or without chemotherapy. 

The sample size was calculated with reference to Sudha et al. [18] reference taking alpha error as 0.05 and power to be 80%, which was calculated as 35 in each arm.

The treatment planning process involved the following steps.

Patient treatment position, immobilization, and planning imaging

The patient was positioned supine on a breast board with a 15-degree tilt. The arms were retracted to the back, and the head was tilted to the opposite side. Three radio-opaque fiducials were kept for scan isocentre localization. Volumetric non-contrast CT scan from lower body of mandible to L3 vertebrae in 2.5mm thickness on a GE Optima CT 580W machine in treatment position was taken involuntary deep inspiratory breathing motion. CT images were transferred to Monaco version 5.11.03 © 2019 Elekta, Inc. three-dimensional treatment planning system (3DTPS).

Target volume delineation and OAR delineation

Target volume contouring - RTOG contouring guideline [21] was followed to create all volumes. The planning target volume (PTV) was generated by giving a 5mm margin in all directions, and the contour was cropped 2 mm from the skin surface. OAR contouring - the OARs for the study included the heart, ipsilateral and contralateral lung, spinal cord.

Dose prescription

Prescription dose for PTV was 50 Gray in 25 fractions, five fractions per week over five weeks, and dose-volume constraints applied for OARs were as follows. The treatment plan was to be accepted if a) 95% of dose covered >= 95% of PTV. If not achieved, at least 90% of the dose covered >= 95% of PTV. b) The volume of the ipsilateral lung being radiated to a dose of 20 Gy was to be kept ≤ 35% (i.e., V_20Gy_ ipsilateral lung ≤35%) [22]. If the optimizing constraints were not achieved, we went with the mean dose to be kept <17 Gy [18]. c) The volume of heart obtaining 25 Gy was tried to be kept ≤10% (i.e., V_25Gy_ Heart ≤10%). If not achieved, the mean heart dose was kept below 15Gy. d) Spinal Cord D_max_ (maximum point dose) <45 Gy.

Forward planning (3D-CRT)

The plan was created using a mono-isocentric technique. The chest wall was irradiated using two opposing tangential beams. Dose prescription was done to isocentre. To ensure adequate PTV coverage and reduce hotspot to less than 107%, the Field-in-Field technique with MLCs was used with multiple subfields. The dynamic weightings used in this study were chosen to maximize PTV dose coverage and homogeneity [14]. A single anterior field with an angle of 10° away from the spinal cord was used to treat the SCF and axillary apex. The prescription was given at the point for the nodal dose but was normalized to reduce hotspots to less than 107% isodose line. The algorithm used was Collapsed Cone and 6 MV photons with a dose rate of 600 MU/min were used for the chest wall, whereas 10MV energy was used for nodal areas.

Inverse planning (IMRT)

A 5F-IMRT plan utilized two opposed tangential beams again with almost similar gantry angles as 3D-CRT-FinF. The gantry angles were 290°(range - 285°-310°), 340° (range - 330-350), 145° (140-150), 115° (110°-115°), 15°. The plans were created by utilizing an inverse planning system in its entirety and 30 points controlled each segment. The moving speed of the multileaf collimator was variable. The doses were calculated with the Monte Carlo algorithm and optimized with the fluence optimizer algorithm using a 5.0 mm grid. The plans were delivered with a 600 MU/min dose rate using the sliding window technique.

VMAT plan creation

The VMAT plans comprised of two optimized coplanar partial arcs (2P-VMAT), with beam-on gantries rotating clockwise from 180° to 292.8° through 90° and 0°. All plans were collimated at a 0° angle. The chest wall and supraclavicular fossa were treated using the same arc plans, and both utilized identical gantry angles. 150 control points were used to create each arc with a 5.0 mm grid. The Monte Carlo algorithm was used to optimize the 2P-VMAT plans.

Plan evaluation and improvement

**Figure 1:**
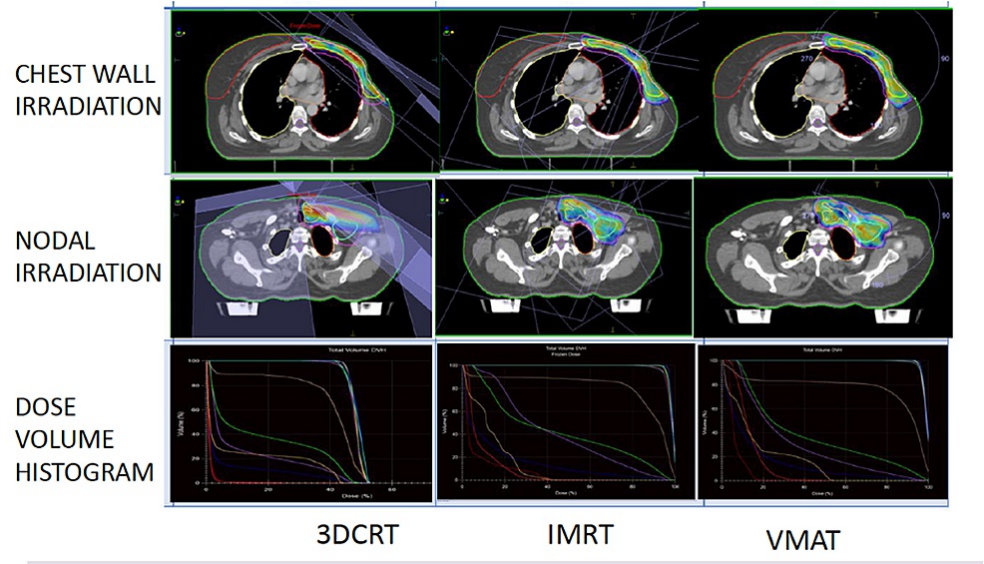
Plan creation and comparison of plans

Evaluation of plan (dose-volume histograms, isodose display, 3-D isodose display) as in Figure 1 and modification was done until the plan was found to be the best possible iteration. The patient's position was verified, and isocentre placement on the treatment machine was done using onboard CT and planning CT. Periodic imaging verification checks were done weekly during treatment. All plans were normalized to the 95% isodose line encompassing 95% of the PTV (V_95%_ =47.5 Gy).

Tabulation of dosimetric data

In the PTV, the dose of 98%, 50%, and 2% of the volume (D_98%_, D_50%_, and D_2%,_respectively) was recorded. The fraction of the PTV getting more than 107% of the prescribed dose (V_107%_) was obtained from DVH and tabulated. The HI was found using ICRU 83 formula: HI = (D_2%_-D_98%_)/D_50%_. The CI was evaluated using ICRU 62 formula: CI = V_47.5_/PTV [23]. Integral dose (ID) = V_Body_*D_Mean_ in the body outside PTV as Joule was obtained and recorded. Dose-volume histograms of all three modalities were generated separately. The mean dose, V_5Gy_, V_10Gy_, V_20Gy_, and V_25Gy_, were recorded for the left lung and the heart. The mean dose to the right lung was also recorded. The images of all plans were compared as in Figure 1.

Statistical analysis

It was done on IBM SPSS (Statistical Package for Social Sciences) version 23 (IBM Corp., Armonk, NY). The data on 3D-CRT, IMRT, and VMAT were expressed as mean with standard deviation. The comparison of the difference in parameters between 3D-CRT, IMRT, and VMAT was carried out using the Kruskal Wallis ANOVA test. The dosimetric profiles of the PTV and OAR were expressed as frequencies and percentages and were compared using the Chi‑square test. All statistical analyses were done at a 5% significance level, and a value of p<0.05 was deemed statistically significant.

## Results

Demographic profile of patients

The mean age of patients was 50; the median age was 49 years (range - 30 to 72). 74.28% of the patient were less than 50 years, and the rest were more than 50 years. The median tumor size was 4.00 cm (1.4-7.2cm). The most common T stage was T2 (35%), followed by T4b (31.42%). N1 (62.85%) was the most common nodal stage, followed by N2a. All patients were non-metastatic (M0). The most frequently encountered stage encountered was stage IIIB- 42.9% followed by Stage IIB - 28.57%. Other demographic parameters are depicted in Table 1.

**Table 1:** Demographic characteristics

S. No	Variable	(N=35)	No of patients (Percentage)
1.	Age (in years)	<=40 years	8 (22.85%)
		40-50 years	18 (51.43%)
		>50 years	9 (25.72%)
2.	Comorbidities	None	21 (60%)
		Hypertension	4 (11.44%)
		Diabetes Mellitus	3 (8.57%)
		Chronic Kidney Disease(CKD)	1 (2.83%)
		Asthma	2 (5.6%)
		Hypothyroidism	2(5.6%)
		Vitiligo	1 (2.83%)
3.	T stage(according to AJCC 8^th^edition)	T2	15(42.85%)
		T3	7(20%)
		T4a	0(0%)
		T4b	11(31.43%).
		T4c	2(5.72%)
		T4d	0(0%)
4.	N stage (according to AJCC 8^th^ edition)	N0	1(2.85%)
		N1	17(48.57%)
		N2a	6(17.14%)
		N2b	5(14.28%)
		N3a	2(5.72%)
		N3b	3(8.58%)
		N3c	1(2.86%)
5.	Stage wise distribution	IIB	10(28.58%)
		IIIA	7(20%)
		IIIB	15(42.85%)
		IIIC	3(8.57%)
		IV	0(0%)
6.	Estrogen receptor	Positive	13(37.15%)
		Negative	22(62.85%)
7.	Progesterone receptor	Positive	21(60%)
		Negative	14(40%)
8.	Her2neu receptor	Positive	11(31.43%)
		Equivocal	2(5.71%)
		Negative	22(62.86%)
9.	Neoadjuvant therapy	Received	25(71.43%)
		Did not receive	10(28.57%)
10.	Adjuvant chemotherapy	Received	33(94.29%)
		Did not receive	2(5.71%)
		Her2 positive received anti her2neu therapy	10(28.57%)

Dosimetric parameters of PTV

**Figure 2:**
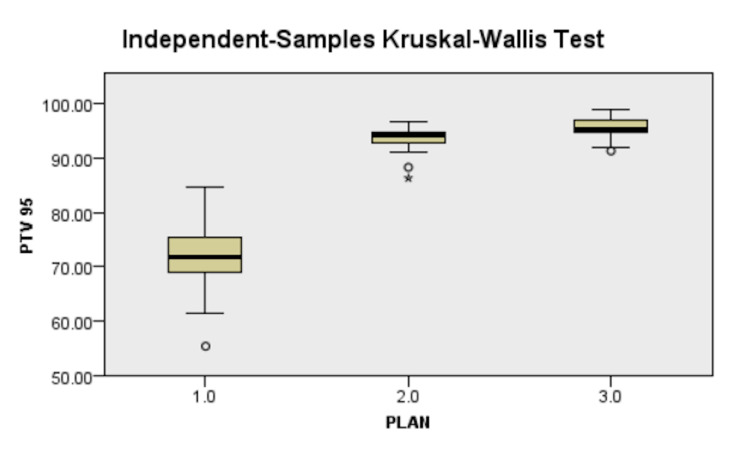
Difference in PTV 95% between plans, 1.0 - 3D-CRT, 2.0 - IMRT, 3.0 - VMAT 3D-CRT - three-dimensional conformal radiotherapy, IMRT - intensity-modulated radiotherapy, VMAT - volumetric-modulated arc therapy

The mean PTV Volume was 847.27±227.71cc (range - 421.91cc - 1371.10cc). The mean volume of patients was 15,272.40±3,920.92 cc. The results are presented in Table 2. IMRT and VMAT led to better V_95%_ of PTV coverage (15% in VMAT and 13% in IMRT) compared to 81.40±6.27% in the 3D-CRT arm (Figure 2). On pairwise analysis, the results were statistically significant, with both IMRT and VMAT being better than 3D-CRT and VMAT being statistically better than IMRT (p=0.037).

**Table 2:** PTV parameters 3D-CRT - three-dimensional conformal radiotherapy, IMRT - intensity-modulated radiotherapy, VMAT - volumetric-modulated arc therapy, PTV - planning target volume

PARAMETER	3D-CRT		IMRT		VMAT		P-value		
	MEAN	SD	MEAN	SD	MEAN	SD	3D-CRT- IMRT	3D-CRT-VMAT	IMRT-VMAT
D_98%_(GY)	40.63	3.22	45.69	0.81	46.42	0.74	0.000	0.000	0.116
D_50%_(GY)	47.97	2.02	49.80	0.28	49.99	0.32	0.000	0.000	0.839
D_2%_(GY)	51.67	2.24	51.89	0.32	51.93	0.33	0.000	0.017	1.000
D_95%_(%)	81.40	6.27	93.70	2.16	95.63	1.82	0.000	0.000	0.037
D_90%_(%)	90.80	2.17	98.65	0.76	98.96	1.47	0.00	0.00	0.316
V_47.5GY_(CC)	540.91	106.20	769.12	212.46	805.32	219.28	0.000	0.000	0.037
CI	0.66	0.11	0.91	0.06	0.96	0.02	0.000	0.000	0.019
HI	0.23	0.05	0.12	0.02	0.11	0.02	0.000	0.000	0.097
HOTSPOT-V_107%_(%)	0.17	0.13	0.01	0.01	0.03	0.03	0.05	0.05	0.05

**Figure 3:**
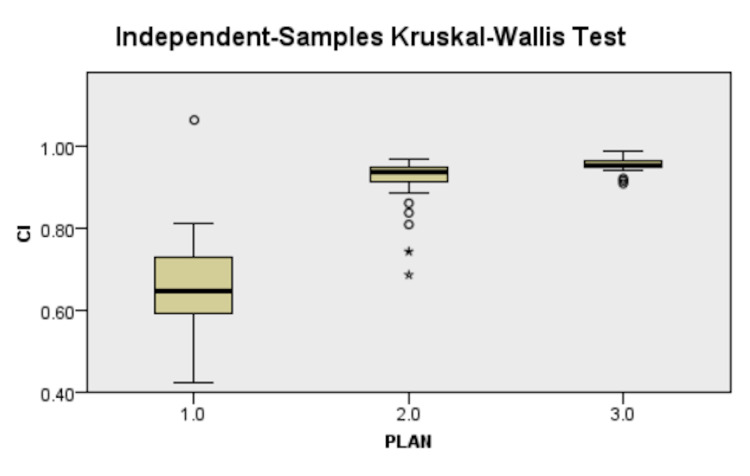
Difference in Conformity index between plans, 1.0 - 3D-CRT, 2.0 - IMRT, 3.0 - VMAT 3D-CRT - three-dimensional conformal radiotherapy, IMRT - intensity-modulated radiotherapy, VMAT - volumetric-modulated arc therapy

**Figure 4:**
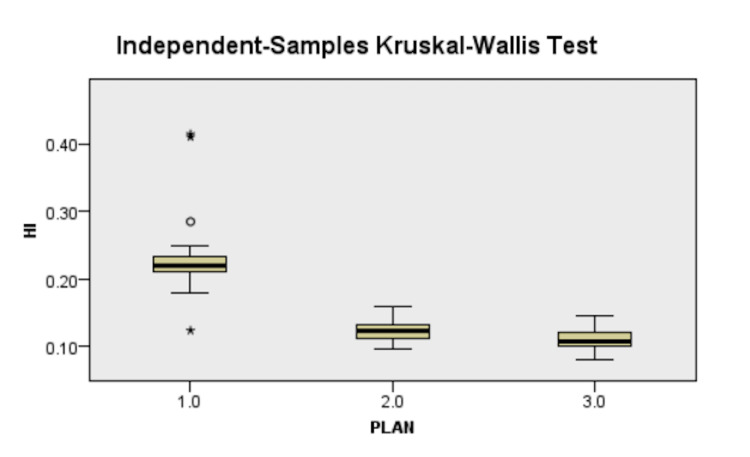
Difference in homogeneity index between plans, 1.0 - 3D-CRT, 2.0 - IMRT, 3.0 - VMAT 3D-CRT - three-dimensional conformal radiotherapy, IMRT - intensity-modulated radiotherapy, VMAT - volumetric-modulated arc therapy

There was better conformity with IMRT (27% increase) and VMAT (30%increase) as compared to 3D-CRT (Figure 3). While both the inverse planning techniques were more homogenous (10% increase) and had lesser hotspots (14% lesser) than 3D-CRT, finding a significant difference was not possible (Figure 4). The results are depicted in Table 2.

Dosimetric parameters of ipsilateral lung

**Figure 5:**
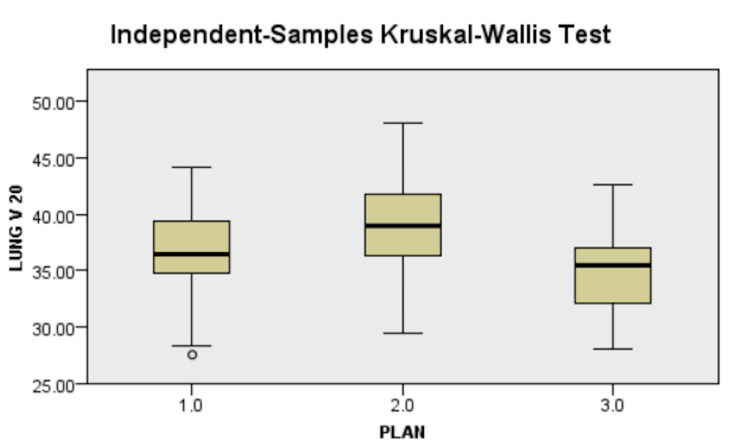
Difference in lung V20 between plans, 1.0 - 3D-CRT, 2.0 - IMRT, 3.0 - VMAT 3D-CRT - three-dimensional conformal radiotherapy, IMRT - intensity-modulated radiotherapy, VMAT - volumetric-modulated arc therapy

Danish Breast Cancer Guideline's recommendations for ipsilateral lung dose were adopted as limiting constraint (V_20Gy_<35%) as supraclavicular irradiation was planned for all patients, and it was challenging to achieve V_20Gy_ <30% as per Graham et al. to achieve at least 95% PTV coverage. Secondary criteria of mean lung dose <17Gy were kept to be clinically accepted. The results are presented in Table 3 and Figure 5.

**Table 3:** Ipsilateral lung dosimetry parameters 3D-CRT - three-dimensional conformal radiotherapy, IMRT - intensity-modulated radiotherapy, VMAT - volumetric-modulated arc therapy

PARAMETER	3D-CRT		IMRT		VMAT		P-value		
	MEAN	SD	MEAN	SD	MEAN	SD	3D-CRT- IMRT	3D-CRT-VMAT	IMRT-VMAT
D_MEAN_(GY)	16.98	2.00	20.06	1.61	18.48	1.55	0.00	0.002	0.002
V_5GY_(%)	53.75	6.73	82.14	9.27	83.96	6.99	0.005	0.000	0.001
V_10GY_(%)	43.55	4.82	58.45	6.05	55.00	6.58	0.004	0.001	0.34
V_20GY_(%)	36.80	4.24	37.64	4.45	35.03	4.20	0.006	0.006	0.006
RIGHT LUNG MEAN (GY)	0.89	0.36	3.73	1.48	6.63	1.13	0.000	0.000	0.000

Dosimetric parameters of heart

**Figure 6:**
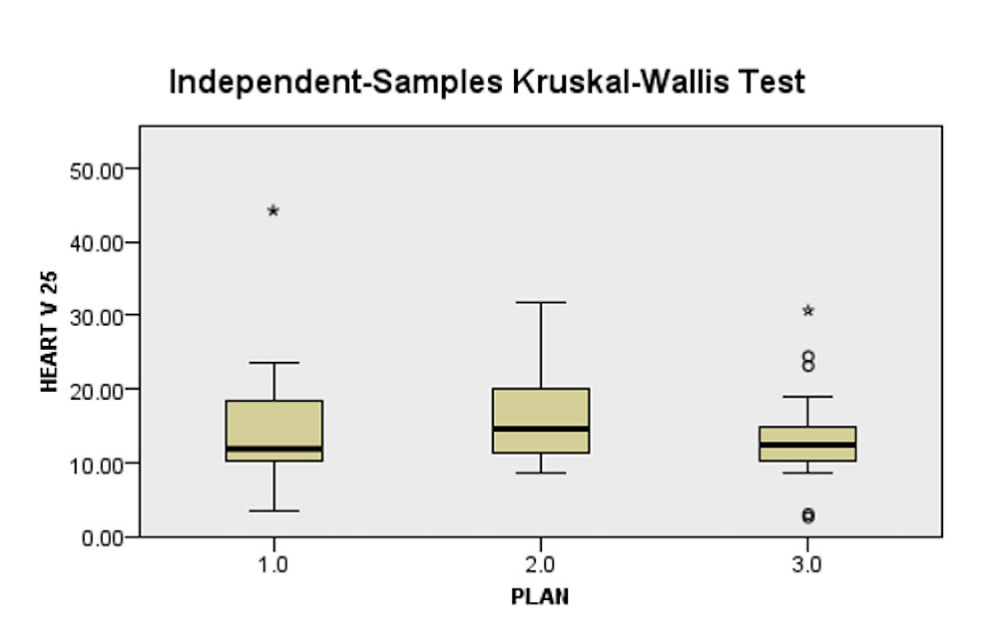
Difference in heart V25 between plans, 1.0 - 3D-CRT, 2.0 - IMRT, 3.0 - VMAT 3D-CRT - three-dimensional conformal radiotherapy, IMRT - intensity-modulated radiotherapy, VMAT - volumetric-modulated arc therapy

V_25Gy_ and D_Mean_ assessed the heart dose in this study, while V_5Gy_ and V_10Gy_ evaluated the low dose area. V_25Gy_ <10% was used as the primary constraint for plan approval as per Gagliardi et al. [24]. In case of the plan is not meeting the primary constraint, a secondary constraint of D_Mean _<15Gy was utilized for plan approval. The results are presented in Table 4 and Figure 6.

**Table 4:** Heart dosimetry parameters 3D-CRT - three-dimensional conformal radiotherapy, IMRT - intensity-modulated radiotherapy, VMAT - volumetric-modulated arc therapy

PARAMETER	3D-CRT		IMRT		VMAT		P-value		
	MEAN	SD	MEAN	SD	MEAN	SD	3D-CRT- IMRT	3D-CRT-VMAT	IMRT-VMAT
D_MEAN_(Gy)	11.89	3.29	14.25	4.86	12.35	3.55	0.003	1.000	0.048
V_5GY_(%)	30.86	9.74	82.76	12.01	89.40	9.48	0.00	0.00	0.00
V_10GY_(%)	25.13	8.97	57.39	15.36	53.94	14.39	0.005	0.000	0.001
V_20GY_(%)	19.71	7.43	25.91	7.32	20.74	8.17	0.005	1.000	0.029
V_25GY_(%)	13.46	5.24	13.15	2.62	12.12	3.22	0.601	0.502	0.602

Dosimetric parameters of miscellaneous structures

The monitor units delivered were highest in the IMRT group followed by VMAT then 3D-CRT. Thus, VMAT led to a shorter treatment time than IMRT. Both the inverse planning methods led to increases in dose to the contralateral breast and the integral dose. Results are presented in Table 5.

**Table 5:** Miscellaneous structure dosimetry parameters 3D-CRT - three-dimensional conformal radiotherapy, IMRT - intensity-modulated radiotherapy, VMAT - volumetric-modulated arc therapy

PARAMETER	3D-CRT		IMRT		VMAT		P-value		
	MEAN	SD	MEAN	SD	MEAN	SD	3D-CRT- IMRT	3D-CRT-VMAT	IMRT-VMAT
MONITOR UNIT	229.44	41.00	793.82	97.10	946.05	139.66	0.000	0.000	0.008
MEAN RIGHT BREAST (GY)	1.19	0.68	2.87	1.19	3.71	0.73	0.000	0.000	0.046
DOSE MEAN (BODY-PTV) [GY]	5.47	0.72	7.41	1.02	7.81	0.83	0.000	0.001	1.000
INTEGRAL DOSE (J)	82.75	19.22	113.68	31.82	119.38	29.49	0.00	0.001	1.000

## Discussion

With most of the population presenting as LABC to the OPD in a developing country like India, a radiotherapy is an indispensable tool for preventing local recurrence. Thus having better tumor control with reduced normal tissue complication rate with better planning techniques was the purpose of this study. Both IMRT and VMAT led to better V_95%_of PTV coverage in IMRT and VMAT compared to 3D-CRT (Figure 2). While Ma et al. [15] found that IMRT had a better dosimetric profile than 3D-CRT and VMAT, their results showed that VMAT led to a better PTV coverage than IMRT and 3D-CRT in that order. Our results are in accordance with Sudha et al. [18] who found a 2% increase in VMAT coverage﻿ (98.21±1.79) versus 3D-CRT (96.30±2.62, p<0.001). The increased number of patients could explain the more considerable difference in our study.

The V_47.5Gy_ mean for our study was 83.84±0.59% for 3D-CRT, 90.78±0.73% in the IMRT arm, and 95.05±0.96% for the VMAT arm. Sudha et al. [18] depicted percentage V_95%_ values of 98.21±1.79% for the 3D-CRT arm and 96.30±2.62% for the VMAT arm. Rastogi et al. [20] have published values of 98±2% for the IMRT arm.

The CI for this study was increased in IMRT (p<0.05) and VMAT plans (p<0.05) compared to 3D-CRT. On pairwise analysis, there was also a statistically significant difference between IMRT and VMAT (Figure 3). A more excellent CI value, ranging from 0 to 1, indicates greater conformity. Hence, VMAT led to the most conformal planning result than IMRT and significantly better than 3D-CRT. Sudha et al. [18] found CI for VMAT (0.97±0.017) to be higher as compared to 3D-CRT (0.95±0.025). Ma et al. [15]. found CI values of ﻿0.64±0.07 and 0.68±0.07 for IMRT and VMAT, respectively. The difference in CI was also evident by reviewing DVH of all plans separately (Figure 1), which showed greater conformality of the 95% isodose color-wash to the PTV in IMRT and VMAT compared to 3D-CRT.

The HI for this study was 0.23±0.05 for 3D-CRT, which was reduced to 0.12±0.02 for IMRT and 0.11±0.01 for VMAT. On pairwise analysis, both IMRT and VMAT were statistically more significant than 3D-CRT, but there was no statistically significant difference between IMRT and VMAT (Figure 4). A lower HI means better homogeneity, thus implying that IMRT and VMAT led to a more homogenous dose distribution than 3D-CRT. A more homogenous dose distribution translates to a better cosmetic outcome and a significant reduction in acute skin reaction incidence, as proposed by ﻿Zaghloul et al. [25]. In comparison, Sudha et al. [18] gave the value of HI for VMAT as ﻿0.23±0.105 and 0.16±0.075 for 3D-CRT. They accounted for VMAT as more inhomogeneous than 3D-CRT due to the thin chest wall, which led to difficulty achieving the tighter dose constraints.

While trying to achieve more excellent PTV coverage, there was a statistically significant greater hotspot in the 3D-CRT arm (0.17±0.53) as compared to the IMRT arm (0.12±0.02) and VMAT arm (0.11±0.02). While Sudha et al. [18] have commented only upon the mean hot spot dose, Ma et al. [15] have kept the criteria of V_110%_ as their criteria, which were 4.26±3.73 for 3D-CRT, ﻿0.22±0.4 for IMRT, and ﻿2.09±3.38 for the VMAT arm.

OAR doses and importance

As far as dose to the OARs is concerned, it was to adhere to the specified constraints as far as possible when achieving better PTV coverage.

Lung Dosimetry

There was a statistically significant difference in left lung V_20Gy_ and left lung mean dose of all three arms on pairwise analysis, with the highest values being found in IMRT followed by VMAT and 3D-CRT in that order (Figure 5). The V_20Gy_ values of VMAT (35.03±4.20) were higher than those of Sudha et al. [18], which was 24.42±3.77, and those of IMRT(37.64±4.45) was higher than that of Rastogi et al. [20] as 22.09±3.89 while all 3D-CRT values were comparable across all studies. The results of this study were similar to the 3D-CRT arms (31.36±6.04) and VMAT arms (34.08±7.16) of Ma et al. [15]. The mean lung dose of this study showed a statistically significant difference between all three arms on pairwise analysis with IMRT (20.06±1.61) having a higher mean lung dose than VMAT (18.48±1.55) and also 3D-CRT (16.98±2.00), showing the lowest mean lung dose among the three arms. The doses here are comparable to values shown by Sudha et al. [18] in 3D-CRT (17.92±1.89) and VMAT arm (17.08±2.46), while the values demonstrated by Rastogi et al. [20] were relatively lower than those found in this study in the IMRT arm (11.39±2.4). In contrast to our results and the above two studies, Ma et al. [15] found no significant difference between IMRT and 3D-CRT but increased mean lung dose by VMAT compared to 3D-CRT and IMRT pairwise analysis.

**Figure 7:**
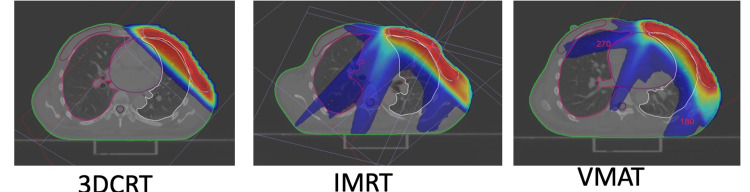
Low-dose irradiated volume (blue line represents the 5Gy isodose volume)

The low dose volume of lung irradiated as depicted by V_5Gy_ and V_10Gy_ in this study were 53.75±6.73 and 43.55±4.82, respectively, for the 3D-CRT arm; 82.14±9.27 and 58.45±6.05, respectively, for the IMRT arm and 83.96±6.99 and 45.00±6.58, respectively, for the VMAT arm (Figure 7). Thus it can be seen that 3D-CRT led to significantly lesser low dose volume for the left lung than IMRT and VMAT, which had significant spillover. It was seen that there was no significant difference between the values in 3D-CRT arms as compared to other studies; there was a lesser dose in the IMRT arm of a study published by Rastogi et al. [20], while the results in this study's VMAT arm were similar to values published by Sudha et al. [18] in their VMAT arm.

There was an effort to minimize the dose to the contralateral right lung to as low as reasonably achievable. It was found that the dose to the contralateral lung was 0.89±0.36 in the 3D-CRT arm, 3.73±1.48 in the IMRT, and 6.63±1.13 in the VMAT arm. The low dose in the 3D-CRT arm may be explained by the angle of the tangential beams, which has very low dose spillage to the contralateral lung, and the fact that was owing to lower PTV coverage, there was decreased dose contralateral lung as well. With IMRT planning the splitting of beams from different angles, there was an increase in PTV coverage, resulting in increased dose entry to the contralateral lung. With VMAT planning, the arc of the beam resulted in a significant exit dose through the contralateral lung.

Heart Dosimetry

The mean heart dose values in this study (Figure 6) were higher than those in the corresponding arms of Rastogi et al. [20], which were 8.96±1.03 in the 3D-CRT arm, ﻿4.57±1.52 in the IMRT arm, and lesser than that of the corresponding component of Sudha et al. [18] which were 15.78±47 in the 3D-CRT arm and 12.86±3.3 in the VMAT arm. Compared to Ma et al. [15], this study had larger mean values in all the arms. The V_25Gy_ values achieved in the above studies have not been explicitly mentioned, most likely to the difficulty in attaining the constraint. The V_20Gy_ values of this study were quite similar to those of the IMRT arm of Rastogi et al. [20] at 22.09±3.89 and the VMAT arm (12.79±6.69) of Sudha et al. [18] with similar results in the 3D-CRT arms of both studies.

In Sudha et al. [18], ﻿the mean values of Heart V_5Gy_ and V_10Gy_ were 39.36±12.72 and 33.56±9.8 for the 3D-CRT plans and 76.18±24.18 and 41.2±16.06 in the VMAT plans, respectively.

Tumor control probability (TCP) was 100% in all arms [26], and 1% normal tissue control probability (NTCP) was estimated for lungs ﻿using the Lyman-Kutcher-Berman Probit model [27] as well as heart [28] in all arms. Hall et al. have postulated that while inverse-planned IMRT enabled formulating a plan that met heart and lung dosage limits, it generally came at the expense of delivering low dose radiation to greater quantities of normal healthy tissue [29]. This hypothesis was substantiated with the results of this study and others before it [15,18,20], which showed statistically significant increased low dose volume for both lung and heart in IMRT and VMAT, as shown in Figure 4. While some have commented upon the likelihood of development of secondary cancer owing to low dose irradiation of lung, heart, and contralateral breast, others like Stovall and Berrington et al. [7] in their studies have refuted the role of radiation in the formation of secondary cancer.

While authors have commented, “Overall, no one breast modulation method (forward or inverse) has been shown to have a clear dosimetric or clinical advantage over another” [30], this study demonstrates better PTV coverage, better conformality, better homogeneity with inverse planning methods at the expanse of increased low dose spillage to heart, both lungs and the contralateral breast.

The study's design was not made to assess the superiority of any modality over others. Further superiority trials are needed to determine a clear winner among the various modalities. Authors like Ma et al. [15] account for respiratory motion by adding a greater PTV margin of 7mm than the 5mm used for this study. The more considerable PTV margin will account for more significant lung and heart volumes irradiation. This differential margin of 3D-CRT and inverse planning methods like IMRT and VMAT was not used here.

The clinical impact of various modalities and toxicity profiles was not possible as a single modality treated all patients. A randomized trial with patients treated by each modality could help evaluate the clinical profile and dosimetric advantage. While better target coverage should lead to a survival advantage, this study was not equipped to assess the impact of treatment delivery on survival as 3D-CRT treated all patients. A longer follow-up would be required to evaluate the effects of inverse planning on survival compared to conformal techniques.

## Conclusions

Both VMAT and IMRT fared better than the standard method as far dosimetry of high dose volumes was considered. Inverse planning methods that worsened performance were the low dose irradiation of the heart, lung, contralateral breast, and integral dose to the body. It would be prudent to conclude that VMAT was superior to IMRT if only target volume and high dose irradiation were prioritized but fared worse when low dose irradiation was brought into the picture. Thus, further studies are needed to determine the dosimetrically superior inverse planning method, acceptable low dose constraints for the inverse planning methods, and the integration of motion management techniques in addition to inverse planning methods.
